# Hyposplenism revealed by *Plasmodium malariae* infection

**DOI:** 10.1186/1475-2875-12-271

**Published:** 2013-08-02

**Authors:** Benjamin Hommel, Alexandre Galloula, Anne Simon, Pierre Buffet

**Affiliations:** 1 Parasitology-Mycology Laboratory, Assistance Publique Hôpitaux de Paris, Groupe Hospitalier Pitié-Salpêtrière, 47 Boulevard de l'Hôpital, 75651, Paris, Cedex 13, France; 2 Hôpital Pitié-Salpêtrière, Service de Médecine Interne, Boulevard de l'hôpital, 75651, Paris, Cedex 13, France; 3 INSERM, UMR-S 945, Paris, France

**Keywords:** Hyposplenism, *Plasmodium malariae*, Howell Jolly bodies, Sickle cell syndrome, Malaria

## Abstract

**Background:**

Hyposplenism, due to splenectomy, inherited red blood cell disorders or acquired conditions such as celiac disease, has an important impact on the severity of malaria, especially in non-immune patients. Conversely, that malaria may reveal functional hyposplenism has not been described previously.

**Methods:**

A 31-year old gardener was diagnosed with an uncomplicated attack of *Plasmodium malariae* 11 years after leaving the endemic area. In addition to trophozoites and schizonts, thick and thin smears also showed Howell-Jolly bodies, pointing to functional hyposplenism. This was later confirmed by the presence of a calcified spleen in the context of S/β + sickle-cell syndrome in a patient previously unaware of this condition.

**Conclusion:**

Malaria may reveal hyposplenism. Although Howell-Jolly bodies are morphologically similar to nuclei of young *Plasmodium* trophozoite, distinction on smears is based on the absence of cytoplasm and irregular size of Howell-Jolly bodies. In the patient reported here, hyposplenism was revealed by the occurrence of *P. malariae* infection relatively late in life. Timely diagnosis of hyposplenism resulted in the implementation of appropriate measures to prevent overwhelming infection with capsulated bacteria. This observation highlights the importance of diagnosing hyposplenism in patients with malaria despite the morphological similarities between ring nuclei and Howell-Jolly bodies on thick smears.

## Background

Hyposplenism corresponds to the impairment of splenic function. This is a serious condition because the spleen plays a major role in immunological and mechanical defences against infections. Diagnosis of spleen dysfunction is based on the measure of its filtering function. The gold standard is the measure of phagocytosis of colloid particles in the spleen using radioisotopic methods. However, quantification of morphological alterations or deformability [1] of circulating red blood cells is a more practical marker [2]. Howell-Jolly bodies are small nuclear remnants from erythroblasts that cannot be removed from red blood cells if the spleen is absent. In sickle cell disease, there is a strong correlation [3] between the number of red blood cells harbouring Howell-Jolly bodies, or the number of “pitted cells” (i e, red blood cells containing small vesicles) in circulation and the intensity of functional hyposplenism. In *Plasmodium* infections, the spleen contributes to innate resistance and limits the magnitude of parasitaemia. This is particularly well established in *Plasmodium falciparum* infection [4]. For other species, such as *Plasmodium malariae*, the influence of hyposplenism on the outcome of infection is poorly documented. Here, is the clinical history report of a patient in whom chronic infection with *P. malariae* (11 years after leaving the endemic area) revealed functional hyposplenism and a major sickle cell syndrome.

## Methods

A 31-year-old gardener was admitted to an emergency department in December 2010. He had moved from Mali to France in 1999 and had never travelled back to Africa or to any tropical country. The patient had a surgical history of inguinal abscess. He had no medication, smoked tobacco for 14 years and did not drink alcohol. Between 2006 and 2009, he experienced episodes of chronic fever and asthenia. On physical examination the patient was febrile with a temperature of 39°C had a 119-beat per minute pulse and a blood pressure of 147/97 mmHg. He reported headache, myalgia and lumbar pain. The rest of physical examination was normal.

The patient was admitted to the internal medicine department. He mentioned repeated flu-like syndromes in the previous three months and recent episodes of rhythmic fever occurring once every three days. At admission, physical examination was unremarkable and main laboratory parameters were: leucocytosis, white blood cells 11.9 × 10^9^/l N (4.0-10.0), microcytic anaemia with haemoglobin 11.5 g/dl N (13.0-17.5) and mean corpuscular volume 72 fl N (80–100), platelets 252 × 10^9^/l N(150–400), creatinaemia 81 μmol/l N (62–106), and inflammatory syndrome with C-reactive protein 33 mg/l N(<5) and ferritinaemia 2,020 μg/l N (30–300).

**Figure 1:**
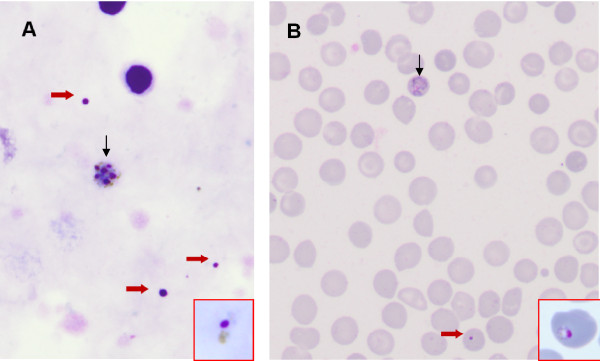
**Diagnosis of *Plasmodium malariae *infection and presence of Howell-Jolly bodies.** Thick **(A)** and thin **(B)** smear at admission (original magnification ×1000, Giemsa –R staining). In red squares, a sample of *P. malariae* rings showing morphological similarities between ring nuclei and Howell-Jolly bodies. The absence of cytoplasm is key for the differential diagnosis. **A**. Two leukocyte nuclei, the rosette-like aspect (vertical arrow) of the schizont (*Plasmodium malariae*), and several Howell-Jolly bodies (red arrows). **B**. Small size of the erythrocyte hosting a P. malariae gametocyte (vertical arrow) and unifected red blood cells showing microcytosis, anisochromasia, and target aspect.

Thin and thick smears were performed from venous blood. The rosette-like aspect of a schizont (Figure 1A), the small size of the erythrocyte-hosting gametocytes on the thin smear (Figure 1B) and the absence of Maurer or Schüffner dots were highly suggestive of *P. malariae* infection, latter confirmed by a positive polymerase chain reaction with primers specific for *P. malariae*[5]. This diagnosis was consistent with the very long incubation period and the rhythm of fever.

Initially, the small, round, purple bodies on the thick smear were interpreted as the nuclei of young trophozoites (rings), thereby strengthening the diagnosis. However, the absence of cytoplasm in contact with these bodies and their markedly variable size contradicted this interpretation. Study of the thin smear confirmed that the bodies corresponded instead to typical Howell-Jolly bodies [6-8]. The thin smear also showed morphological abnormalities of red cells: microcytosis, anisochromasia, target cells and schistocytes, raising the hypothesis of an associated haemoglobin disorder. Liquid chromatography of haemoglobin revealed a S/β + sickle cell syndrome (HbS 60% and heterozygous beta thalassaemia ß + with HbA2 6.5% HbF 6.1%) [9].

**Figure 2:**
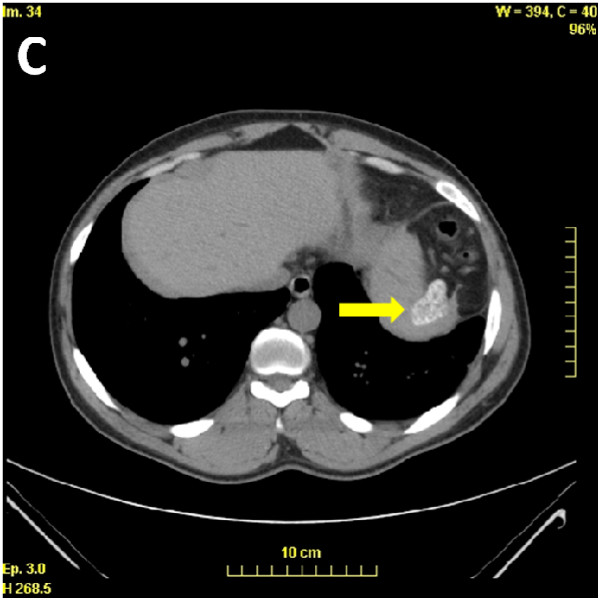
Abdominal CT-scan identify a small calcified spleen (yellow arrow).

The patient had no abdominal surgery scars. Due to the presence of Howell-Jolly bodies and the S/β + sickle cell syndrome, a computed tomography scan of the abdominal region was performed and showed an atrophic calcified spleen (Figure 2).

The malaria attack resolved rapidly with a three-day course of oral chloroquine. Clinical and laboratory markers returned to normal in one week. The patient was discharged after being vaccinated for encapsulated bacteria (*Streptococcus pneumoniae*, *Haemophilus influenzae* and *Neisseria meningitidis*) and provided with long-term oral beta-lactams to prevent potential complications of asplenia [10]. Malaria prophylaxis was recommended in case of travel to Africa.

## Conclusion

In patients with sickle cell anaemia, repeated sequestration crises leading to infarctions generally result in early splenic atrophy with calcifications [11]. This evolution is almost constant in adult patients with homozygous sickle cell disease but is less frequent in patients with the S/β + sickle cell syndrome [11]. Progressive hyposplenism and splenic atrophy in this patient were revealed by a malaria episode, and occurred relatively late in life. Sickle cell trait, i e, the heterozyte form, is protective against severe and fatal *P. falciparum* infections [12], whereas in homozygosity, protection against severe malaria is counterbalanced by hyposplenism which appear during childhood [13,14]. This observation suggests the existence of a similarly balanced influence of the presence of HbS on the evolution of infection with *P. malariae*.

Much less is known on the impact of anatomical or functional hyposplenism on the course of infections with *Plasmodium vivax*, *Plasmodium ovale*, *Plasmodium knowlesi* and *P. malariae* than on the more frequent *P. falciparum *[8,15]. Indeed, *P. falciparum* infection is more frequently severe and fatal in non-immune splenectomized patients [4,8]. *Plasmodium malariae* infection can be severe in patients with splenectomy or functional asplenia [16-18]. Here the patient experienced only mild symptoms. It is assumed that in *P. malariae* as observed with *P. falciparum* patients who acquired immunity before becoming asplenic, uncomplicated attacks are more frequent than severe malaria [4].

Presence of Howell-Jolly bodies on a blood sample is a fairly specific indicator of hyposplenism [19,20]. In this patient their presence on the thick/thin smear was due to sickle cell anaemia but presence of Howell-Jolly bodies is also be associated with several diseases: bone marrow transplantation (40%), celiac disease (33-76%), HIV infection/AIDS 36 (%), alcoholic liver disease (37-100%) and systemic lupus erythematosus (7.5%) [2]. The prevalence of hyposplenism is generally underestimated and the diagnosis often overlooked. This has potentially severe consequences in infectious diseases in general, and in malaria in particular. Detecting hyposplenism can thus be life-saving as it triggers the adoption of appropriate preventive measures against overwhelming infections [2].

This observation highlights the importance of diagnosing hyposplenism in patients with malaria despite the morphological similarities between ring nuclei and Howell-Jolly bodies on thick smears.

## Consent

Consent was granted by patient for the publication of this case report.

## Competing interests

PB is supported by the Bill & Melinda Gates Foundation and SANOFI. BH, AS and AG declare that they have no competing interests.

## Authors’ contributions

BH and PB wrote the manuscript with contribution of AS. AG made the diagnosis. AG and AS were responsible for the patient’s care and treatment and reviewed the manuscript. PB and BH made the microbiological diagnosis. All authors read and approved the final manuscript.

## References

[B1] Prendki V Ndour PA Jais X Ciceron L Settegrana C Buffet P Reduced deformability of circulating erythrocytes: a marker of hyposplenism Am J Hematol 2012 87 E81 E82 10.1002/ajh.23297 22847468

[B2] Di Sabatino A Carsetti R Corazza GR Post-splenectomy and hyposplenic states Lancet 2011 378 86 97 10.1016/S0140-6736(10)61493-6 21474172

[B3] Rogers ZR Wang WC Luo Z Iyer RV Shalaby-Rana E Dertinger SD Shulkin BL Miller JH Files B Lane PA Thompson BW Miller ST Ware RE BABY HUG: Biomarkers of splenic function in infants with sickle cell anemia: baseline data from the BABY HUG Trial Blood 2011 117 2614 2617 10.1182/blood-2010-04-278747 21217080 PMC3062353

[B4] Bach O Baier M Pullwitt A Fosiko N Chagaluka G Kalima M Pfister W Straube E Molyneux M Falciparum malaria after splenectomy: a prospective controlled study of 33 previously splenectomized Malawian adults Trans R Soc Trop Med Hyg 2005 99 861 867 10.1016/j.trstmh.2005.03.008 16099487

[B5] Snounou G Detection and identification of the four malaria parasite species infecting humans by PCR amplification Methods Mol Biol 1996 50 263 291 8751365 10.1385/0-89603-323-6:263

[B6] Schnitzer B Sodeman T Mead ML Contacos PG Pitting function of the spleen in malaria: ultrastructural observations Science 1972 177 175 177 10.1126/science.177.4044.175 4339353

[B7] Robertson DA Bullen AW Hall R Losowsky MS Blood film appearances in the hyposplenism of coeliac disease Br J Clin Pract 1983 37 19 22 6838752

[B8] Buffet PA Safeukui I Deplaine G Brousse V Prendki V Thellier M Turner GD Mercereau-Puijalon O The pathogenesis of *Plasmodium falciparum* malaria in humans: insights from splenic physiology Blood 2011 117 381 392 10.1182/blood-2010-04-202911 20852127 PMC3031473

[B9] Weatherall DJ Genetic variation and susceptibility to infection: the red cell and malaria Br J Haematol 2008 141 276 286 10.1111/j.1365-2141.2008.07085.x 18410566

[B10] Davidson RN Wall RA Prevention and management of infections in patients without a spleen Clin Microbiol Infect 2001 7 657 660 10.1046/j.1198-743x.2001.00355.x 11843905

[B11] Ozgen A Akata D Arat A Ozdogan M Akhan O Ozmen MN Splenic calcifications in heterozygote sickle cell patients Abdom Imaging 1999 24 188 190 10.1007/s002619900473 10024409

[B12] Williams TN Mwangi TW Wambua S Peto TE Weatherall DJ Gupta S Recker M Penman BS Uyoga S Macharia A Mwacharo JK Snow RW Marsh K Negative epistasis between the malaria-protective effects of alpha + −thalassemia and the sickle cell trait Nat Genet 2005 37 1253 1257 10.1038/ng1660 16227994 PMC3521056

[B13] Komba AN Makani J Sadarangani M Ajala-Agbo T Berkley JA Newton CR Marsh K Williams TN Malaria as a cause of morbidity and mortality in children with homozygous sickle cell disease on the coast of Kenya Clin Infect Dis 2009 49 216 222 10.1086/599834 19514855 PMC2727464

[B14] Pearson HA McIntosh S Ritchey AK Lobel JS Rooks Y Johnston D Developmental aspects of splenic function in sickle cell diseases Blood 1979 53 358 365 760858

[B15] Luzzatto L Sickle cell anaemia and malaria Mediterr J Hematol Infect Dis 2012 4 e2012065 23170194 10.4084/MJHID.2012.065 PMC3499995

[B16] Petersen E Hogh B Marbiah NT Hanson AP The effect of splenectomy on immunity to *Plasmodium malariae* and *P. falciparum* in a malaria immune donor Trop Med Parasitol 1992 43 68 69 1598514

[B17] Tapper ML Armstrong D Malaria complicating neoplastic disease Arch Intern Med 1976 136 807 810 10.1001/archinte.1976.03630070051016 1065254

[B18] Toma E Malaria in splenectomized patients Clin Infect Dis 1993 17 936 937 8286651 10.1093/clinids/17.5.936

[B19] Groom AC Schmidt EE MacDonald IC Microcirculatory pathways and blood flow in spleen: new insights from washout kinetics, corrosion casts, and quantitative intravital videomicroscopy Scanning Microsc 1991 5 159 173 discussion 173–154 2052921

[B20] Grover R Wethers DL Spleen dysfunction in hemoglobinopathies determined by pitted red cells Am J Pediatr Hematol Oncol 1988 10 340 343 10.1097/00043426-198824000-00015 3239712

